# Use of complementary and alternative medicine at Norwegian and Danish hospitals

**DOI:** 10.1186/1472-6882-11-4

**Published:** 2011-01-18

**Authors:** Laila J Salomonsen, Lasse Skovgaard, Søren la Cour, Lisbeth Nyborg, Laila Launsø, Vinjar Fønnebø

**Affiliations:** 1 National research center in complementary and alternative medicine, NAFKAM Faculty of health science, University of Tromsø, 9037 Tromsø, Norway; 2 Interdisciplinary CAM-Research at the University of Copenhagen, KUFAB Institute of sociology, Øster Farimagsgade 5, DK - 1014 København K, Denmark; 3 Department of Public Health, University of Copenhagen, Øster Farimagsgade 5, 1014 Copenhagen K, Denmark

## Abstract

**Background:**

Several studies have found that a high proportion of the population in western countries use complementary and alternative medicine (CAM). However, little is known about whether CAM is offered in hospitals. The aim of this study was to describe to what extent CAM is offered in Norwegian and Danish hospitals and investigate possible changes in Norway since 2001.

**Methods:**

A one-page questionnaire was sent to all included hospitals in both countries. The questionnaire was sent to the person responsible for the clinical activity, typically the medical director. 99 hospitals in the authority (85%) in Norway and 126 in Denmark (97%) responded. Given contact persons were interviewed.

**Results:**

CAM is presently offered in about 50% of Norwegian hospitals and one-third of Danish hospitals. In Norway CAM was offered in 50 hospitals, 40 of which involved acupuncture. 19 hospitals gave other alternative therapies like biofeedback, hypnosis, cupping, ear-acupuncture, herbal medicine, art therapy, homeopathy, reflexology, thought field therapy, gestalt therapy, aromatherapy, tai chi, acupressure, yoga, pilates and other. 9 hospitals offered more than one therapy form. In Denmark 38 hospitals offered acupuncture and one Eye Movement Desensitization and Reprocessing Light Therapy. The most commonly reported reason for offering CAM was scientific evidence in Denmark. In Norway it was the interest of a hospital employee, except for acupuncture where the introduction is more often initiated by the leadership and is more based on scientific evidence of effect. All persons (except one) responsible for the alternative treatment had a medical or allied health professional background and their education/training in CAM treatment varied substantially.

**Conclusions:**

The extent of CAM being offered has increased substantially in Norway during the first decade of the 21^st ^century. This might indicate a shift in attitude regarding CAM within the conventional health care system.

## Background

Over the recent decades, a substantial increase in the use of, and change in attitudes towards complementary and alternative medicine (CAM) has been observed in Norway. Medical personnel are generally positive to the use of CAM, nurses and physiotherapists more than physicians [1-3]. Of Norwegian doctors 34-64% recommends or refers their patients to acupuncture [2,4]. Every tenth Norwegian physician has used acupuncture to treat their own health problems [4], while other CAM therapies to a lesser degree are referred to or used [2,5]. In Norway physicians working in hospitals are more sceptical about CAM than general practitioners [1,2]. Nearly every maternity ward has one or more midwives trained in acupuncture. Approximately 50% of the population have used CAM given by a provider outside the health care system or by health personnel inside the health care system 'within the last 12 months'. Nearly one third have received the treatment only from health personnel [6].

In 2001 CAM was offered in about one-fourth of Norwegian hospital units, with a substantial increase during the 1990 s [7]. Within-hospital practice of CAM most often depended upon one person. Interest in establishing collaboration between health care personnel and CAM therapists has been expressed at several Norwegian hospitals [8], and such collaboration has been encouraged by the Norwegian government [9]. Funding has, however, not yet been allocated.

There has been little research in Denmark on attitudes of medical personnel with regard to CAM. A study from 2000 shows that 73% of Danish doctors were positive towards research within CAM, especially in acupuncture and reflexology and if research is based on randomized clinical trials [10]. Two qualitative studies showed that both medical doctors and pharmacists educated in CAM modalities had severe difficulties in practising CAM inside the health care system and had therefore chosen to work in private clinics [11,12]. The 2005 population use of CAM outside the health care system was 22,5% (within the last year) [13].

The reported level of CAM offered in Swiss hospitals is higher when asking at the department level (37%) compared to asking the hospital managers (33%) [14]. In Israel 10 out of 24 public hospitals offer different modalities of CAM. The motivation for doing that is an attempt to gain a larger share of the health care market, and the value of CAM was not considered [15]. The tendency outside of Europe is also characterized by an increasing spread of alternative treatment within the health sector; In an annual survey of hospitals The American Hospital Association (AHA) has since 1999 reported use of alternative treatment in American hospitals. The proportion of hospitals reporting use of alternative treatment has increased from 7.7% in 1999 to 37.7% in 2008 [16]. Little is known beyond these studies about the extent of CAM offered in hospitals in other countries.

The use of CAM is changing and changes are also seen in the attitude to CAM among health care personnel [1-3,13]. In Norway The National Research Center in Complementary and Alternative Medicine (NAFKAM) has decided to monitor changes in the use of CAM in hospitals approximately every 5^th ^year. Our former study has shown to be important both in communication with politicians and in establishing collaborating research partners in hospitals. In the light of differing CAM legislation and regulation in Europe [17], it is important to detect whether the European level of CAM use in hospitals is similar across national boundaries. We invited several European countries to participate in the study, but lack of funding made it impossible, except for Denmark. So far we know little about the effects/side effects and the efficacy/safety of CAM treatments, and it is therefore important to monitor the changes in the use of CAM and thereby justify the need for and use of research funding.

Government initiatives with regard to CAM research in Norway and Denmark differ. In Norway the government has initiated the establishment of NAFKAM, including "The National Information Center for Alternative Treatment", NIFAB. NAFKAM performs and stimulates research in CAM. In Denmark "The Knowledge and Research Center for Alternative Medicine", VIFAB, is an independent institution under the Danish Ministry of Health and Prevention. VIFAB is an information center and administers the allocation of research grants, but does not itself conduct research. In both countries acupuncture given inside the health care system is covered by the national health insurance.

The aim of this study was to describe to what extent CAM is offered in Norwegian and Danish hospitals and investigate possible changes in Norway since 2001.

## Methods

### Organization of the hospitals

Norway has four regional health authorities responsible for the secondary and tertiary health care service. These regional health authorities have in turn established smaller local health authorities that are responsible for one or more hospitals with both somatic and psychiatric units.

The organization of the health care system in Denmark corresponds to the Norwegian, but is divided into five regional health authorities. As in Norway, the local somatic and psychiatric hospitals are run by the health authority in the region.

In addition to this government-owned health care system, both countries have a limited number of small, private hospitals. In both countries some of these have funding contracts with the regional health authorities, others operate independently.

This study includes the 82 public hospitals in Norway as well as the 35 private hospitals with a funding contract with the regional health authorities, altogether 103 somatic hospitals (some also include psychiatric units) and 14 private psychiatric units. In Denmark 99 public hospitals and 31 private hospitals with a funding contract with the regional health authorities are included, altogether 102 somatic and 28 psychiatric hospitals.

### Questionnaire

In February 2008 a one-page questionnaire was sent to all included hospitals in both countries [Additional file 1].

The questionnaire was sent to the person responsible for the clinical activity, typically the medical director of the hospital. He/she was asked to report whether or not one or more specified CAM therapies were offered at the hospital. The given alternatives were: 1 - No CAM therapy is offered, 2 - Acupuncture, 3 - Homeopathy, 4 - Reflexology, 5 - Phytotherapy, 6 - Alternative diet, 7 - Other CAM therapy (which the respondents were asked to specify). For each therapy reportedly offered we requested information about a local contact person. One written reminder was sent in case of no reply, and contact was made by telephone if none of the written requests were answered. The person responsible for the clinical activity was asked to fill in one separate questionnaire for each hospital unit within their health authority. The questionnaire did not include a definition of CAM. This was done to avoid imposing constraints on the reporting of CAM use in the individual hospital.

Information was returned by 99 of 117 hospitals in Norway (84,6%), and 126 of 130 hospitals in Denmark (96,9%). In Norway 97 hospitals returned the questionnaire by mail and 2 provided the information by phone only. In Denmark 115 hospitals returned the questionnaire and 11 provided the information by phone only.

### Interviews

The given contact persons for the therapy at each hospital were in the next phase approached through a structured interview by phone or was given a mail-in questionnaire. In Norway we were able to make a telephone interview with contact persons from 42 of 50 hospitals that reported offering CAM. In Denmark we received completed questionnaires from 25 hospitals and interviewed 7 contact persons by phone, out of the 39 hospitals that reported offering CAM. Some hospitals reported more than one contact person (they had more than one alternative treatment or alternative treatments at several departments.) The total number of contact persons interviewed were 64 in Norway and 38 in Denmark.

Information provided during the interviews were: Year and motive for starting up CAM treatment, patient groups receiving the treatment, clinical indications, background and potential education requirements for the practitioners, ongoing research projects related to the treatment, degree of interest in participating in future research projects within CAM, whether or not the employees were offered CAM, if information about CAM was given to patients on request, and information about the practitioners' own perception of whether they were practicing CAM or not.

### Statistical methods

This is a survey soliciting information from all hospitals in the two countries in a specified year. It is therefore inappropriate to apply formal statistical testing of the results.

### Ethical approval

The study does not include human information or material and did not need any etichal approval.

## Results

### Questionnaire

In Norway 50 of 99 responding hospitals (50,5%) offered CAM. In Denmark, CAM was offered at 39 of 126 responding hospitals (31%).

Table 1 shows for both countries that more somatic than psychiatric hospitals offer CAM, more public than private hospitals offer CAM, and hospitals with more than 100 beds offer CAM more often than hospitals with less than 100 beds. The highest proportion was seen in Norwegian public somatic hospitals with more than 100 beds, of which 58% reported offering CAM.

**Table 1:** CAM on offer in Norwegian and Danish hospitals in number and percentage

	Type of Hospital											
	Somatic*^1^		Psychiatric*^2^		Public		Private		Less than 100 beds		100 or more beds	
	n	%	n	%	n	%	n	%	n	%	n	%
Norway	46/88	52,3	4/11	28,6	41/68	60,3	9/31	29	18/57	31,6	32/42	76,2
Denmark	33/100	33	6/26	23,1	33/98	33,7	6/28	21,4	13/60	21,7	26/66	39,4

*^1^: Some of the Norwegian and Danish somatic hospitals also include psychiatric units.

*^2^: The Norwegian psychiatric hospitals cover only private institutions and for Denmark only public.

### Therapies offered

In addition to the questionnaire information about the specified therapeutic modalities offered, the interviews with contact persons at each hospital resulted in information about additional modalities offered. Reported therapies at the hospitals reportedly offering CAM are summarized in Table 2.

**Table 2:** Therapies offered at Norwegian and Danish hospitals

	Acupuncture		Other CAM modalities		More than one CAM modality offered	
	Number	%	Number	%	Number	%
Norway (N = 50)	40/50	80,0	19/50	38,0	9/50	18,0
Denmark (N = 39)	38/39	97,4	1/39	2,6	None	0,0

Norway: Other CAM therapies include: TENS-trancutan electric nerve stimulation, Biofeedback, hypnosis, cupping, ear-acupuncture, herbal medicine, massage, musical therapy, art- and expression therapy, art therapy, reflexology, thought field therapy, alternative food, gestalt therapy, psychodrama, aromatherapy, tai chi, acupressure, sound- and music therapy, yoga, pilates and foot-massage.

Denmark: Other CAM therapies include: EMDR - Eye Movement Desensitisation and Reprocessing light therapy.

### CAM offered in the different fields of medicine

We did not ask directly for information on the types of departments in which CAM was offered. However, this information was given by the respondent either in the questionnaire or during the interview. The fields of medicine where CAM was offered are shown in Table 3.

**Table 3:** CAM offered in different fields of medicine

	Departments	
	Norway	Denmark
Acupuncture	Maternity ward, emergency medicine dept, dept pain therapy/pain clinic, dept for blood disease, dept of physical/occupational therapy, Psychiatric dept, oncology dept, surgical/anaesthetic dept, gynaecological dept, out-patients' pain clinic, substance abuse-related care, palliative care	Maternity ward, dept pain therapy/pain clinic, dept for blood disease, Psychiatric dept, oncology dept, surgical/anaesthetic dept, gynaecological dept, out-patients' pain clinic, substance abuse-related care, palliative care
Other CAM therapies	Psychiatric dept, patients at different departments, cancer rehabilitation, dept physical/occupational therapy, substance abuse-related care, palliative care, maternity ward, dept reumathology, center for patients education, palliative care	Information not given

**Figure 1:**
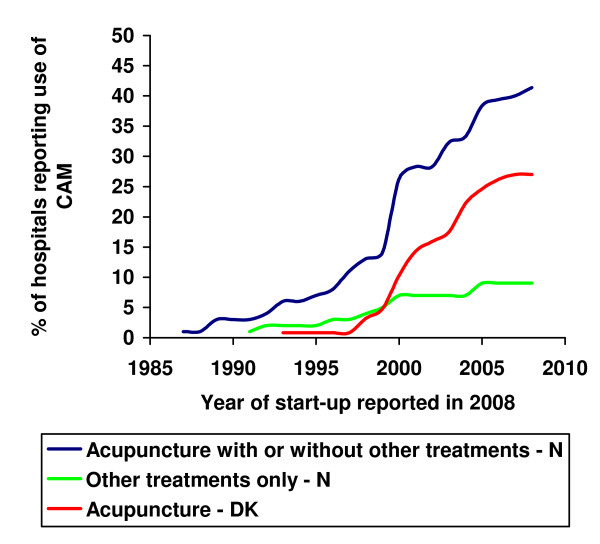
**% of hospitals reporting use of CAM**. Year of start-up for the Norwegian (N) and Danish (DK) hospitals that reportedly offered CAM in 2008. The blue line shows the number of hospitals in Norway that either offer acupuncture or in addition also other treatments. The green line shows the number of hospitals that only give other kind of treatments than acupuncture. Other therapy forms are listed below Table 2. The red line shows the number of hospitals in Denmark that offer acupuncture. Norway, total respondents = 99, Denmark, total respondents = 126.

The temporal increase in CAM offered at Norwegian and Danish hospitals is shown in Figure 1.

### Interview with contact person

Table 4 shows selected data from the interviews on CAM treatments offered. Some of the 64 contact persons in Norway and 38 in Denmark represent the same hospital (some hospitals offered more than one CAM treatment or the same treatment at several departments). Five contact persons from four Norwegian hospitals reported no CAM currently offered when interviewed.

**Table 4:** Selected data from interviews with contact persons

		Norway %/n (total number of contact persons = 64)	Denmark %/n (total number of contact persons = 38)
Year of start-up	Before 2000	45,3%/29	26%/10
	After 2000	46,9%/30	74%/28
	Not given	7,8%/5	0
Motive for starting up	Pain relief:	17%/11	0 ***
	Treatment without side effects:	6%/4	21%/8
	Scientific evidence:	5%/3*	32%/12
	Interest from the employees:	25%/16	21%/8
	Interest from patients:	9%/6	8%/3
	Other:	38%/24**	18%/7**
Ongoing research projects on CAM		9%/6	42%/15
CAM offered to employees		23%/15	59%/23
Information given about CAM		84%/54	74%/28
CAM or not	Yes	36%/23	43%/16
	No	53%/34	57%/22
	Do not know	11%/7	

* The argument of scientific evidence was often used as a reason for why the treatment was not considered as CAM and not as much as a motive for starting up.

** Examples of other motives: "in fashion", extra offer, successful attempt against weight loss/nausea, heard the treatment worked, when nothing else work, good impact on health according to bad sleep/anxiety/immune system/energy, holistic approach.

*** The reason why "pain relief" is not reported as a motive for starting up use of CAM in Danish hospitals might be that it is included in the other four motives. As seen in table 5 pain relief is an important indication for use of acupuncture in Danish hospitals.

As shown in Figure 1 and Table 4 CAM was offered in hospitals earlier in Norway than in Denmark. Table 4 also shows that ongoing research projects on CAM and CAM offered to employees were more common in Danish than in Norwegian hospitals in 2008.

### Other results from the interview

#### Background of the practitioners

In most cases physiotherapists, midwives, physicians and nurses employed at the hospitals provide the CAM treatment in both Norway and Denmark.

One Norwegian hospital has a person with acupuncture and homeopathy competence employed as a "consultant", one uses a free-lance acupuncturist, one has music therapists and a hired yoga-teacher or sometimes yoga instruction was given by an employee. One hospital has a nurse assistant performing aromatherapy. In Denmark four of the contact persons giving the CAM treatment reported having a long acupuncture education, one of them in Traditional Chinese Medicine (TCM).

In Norway those giving acupuncture are either required by the hospitals to be a member of the "Norwegian Acupuncture Association", to have studied acupuncture for 6-9 months, or to be health personnel with a specified acupuncture course (physicians), a two-week course in the theory and practice of acupuncture (midwives), or have competence in at least two acupuncture points (midwives). For art-therapy the practitioner had to have therapy-specific education.

Hospitals in Denmark require the acupuncturists to have educations of different lengths, like courses at "Danish Medical Association of Acupuncture", "The Association of Danish Physiotherapists" or "TCM- Denmark".

#### Indications for offering CAM

As shown in Table 5 the indications for offering acupuncture are similar in the two countries, but a larger variety of indications characterise the Norwegian data in regard to other CAM modalities.

**Table 5:** Indications for offering CAM.

	Indication	
	Norway	Denmark
Acupuncture	**Pain**: Back pain, pain in neck/shoulder/muscles/joints/stomach/pelvic region, headache, migraine, sciatic pain	**Pain**: pelvic pain, muscular pain or chronic pain like sciatic pain,
	**For pregnancy/giving birth**: retended placenta, inadequate milk production, tension, induction of birth, ovum harvesting, nausea, vomiting, infertility, pain	**For pregnancy/giving birth: **nausea, ovum harvesting, infertility, pain
	**Other**: insomnia, arthritis, post-operative and chemotherapy-induced nausea, anxiety, diabetes and relaxation	**Other**: oedema, anxiety, stepping down doses of medicine, chemotherapy-induced nausea
Other CAM therapies	Pelvic tension, colic, anxiety, ADHD, behaviour problems, nausea, substance abuse and allergy	Not answered

## Discussion

The results in this paper are descriptive and they show to what extent CAM is offered in Norwegian and Danish hospitals as well as changes in Norway since 2001. The study shows that one third of the Danish and close to 50% of Norwegian hospitals offer CAM treatment. In addition to acupuncture a broad spectre of alternative therapies were offered in Norway. The study shows a substantial increase in the proportion of Norwegian hospitals offering CAM since 2001.

### Selection bias

The response rates in the present study are high, though slightly lower in Norway than in Denmark. The Danish response rate is at the same level as in the Norwegian study from 2001 [7]. There are no clear reasons for a lower Norwegian response rate in the present study, but it could have been influenced by the re-organization of hospitals into regional health authorities. The short and clear questionnaire has shown to be a strength in the study. Only one minute has been required for the person responsible for the clinical activity to fill it out, and this circumstance has probably been of significant importance for the high response rate. In some cases the person responsible for the clinical activity has been asked to answer on behalf of more than one hospital due to the administrative organization of hospitals. This has not affected the response rate negatively, but the answers will thereby represent fewer people's definition of CAM and their overview of the activities going on at the hospital units.

### Information bias

This study was based on the respondents' definition of CAM and his/her overview of CAM activities within the hospital. In Norway several of the individuals giving the treatment disagreed with the statement that they were practicing CAM. This was also reported in 2001 [7]. This could indicate an overestimation of CAM offered by the persons responsible for the clinical activity. Several contact persons in the Norwegian hospitals gave additional information on CAM offered at other departments within the hospital. For this reason the extent of CAM offered could also have been underestimated. We do not know how many hospitals offering CAM failed to report it. Our total estimate on CAM offered can be assumed to be close to the truth.

### The definition of CAM

When dealing with CAM approaches within the health care system, "complementary and alternative medicine", abbreviated CAM, is the term most often used.

No applicable official definition of CAM exists in Denmark. The Norwegian alternative treatment act of illness etc, 2004, [18] define CAM as; "*health related treatment performed outside of the health service and is not practised by authorized health personnel. Treatment performed inside the health service or by health personnel is included by the concept alternative medicine when methods that normally are practised outside of the health service are used"*. CAM is in some official contexts defined by what it is not (not funded by the public health insurance, not performed within the conventional health system, not documented as effective, not a part of the conventional health care educational system), in other official contexts the definition is based on the persons performing the treatments (how they are educated or professionally organized) [19,20]. We have not found these definitions to provide a useful basis for a study, since the perception of CAM differs within the health care system, and by basing the questionnaire on one definition we would risk incomplete reporting of CAM offered in the hospitals. The study is therefore partly based on the respondents' own definition of CAM as well as his/her overview of CAM activities going on at the different hospital units. Hence the respondents were not given one definition of CAM, but we enclosed a list of the most commonly used CAM therapies.

That CAM is not easily defined is in several ways confirmed by the study. Both the Norwegian study from 2001 and the Swiss study from 2005 showed that employees within the same hospital do not always agree on whether a given treatment modality is alternative or not. After the previous Norwegian study in 2001 was published, the journal received a letter to the editor where the writers expressed disagreement with transcutane electric nerve stimulation (TENS) being classified as CAM [21]. This divergence of opinion is in the present study primarily related to acupuncture. When asking the contact persons, we find that more than half of the contact persons interviewed do not consider acupuncture as CAM. This circumstance could be explained with reference to one official definition of CAM as a treatment modality not performed within the conventional health care system [20,9]. But the disagreement might also indicate a grey area of treatment considered neither as alternative nor as conventional - an area constantly developing and changing. Finally the significance of different types of acupuncture must be taken into consideration. It is possible that differences between western medical acupuncture and traditional eastern acupuncture play a role with regard to the different opinions.

### CAM offered in Norway

The present study shows that CAM is offered at more hospitals in a larger variety of therapies compared to the 2001 study [7]. In 2001 approximately one fourth of the Norwegian hospitals offered CAM, mainly acupuncture. As in 2001, the interest of the health personnel is the main given factor prompting the introduction of CAM therapies. Acupuncture is, however, an exception where the introduction of the therapy is more often initiated by the leadership rather than the employee. In 2001 acupuncture was more often considered as CAM, 57%, compared to the present study, 36%. Compared to the 2001 survey the contact persons in the present study more often refer to scientific evidence as a reason for offering CAM, and scientific evidence is more often used as an argument for why treatments are not considered to be CAM. Both surveys show the same level of demands for education. In the present study the indication for offering CAM has expanded, and a larger variety of educational backgrounds among the practitioners is seen.

### CAM offered in Danish hospitals compared with Norwegian data

The extent of CAM offered and the varieties of CAM modalities on offer in Denmark is at the level of the Norwegian study from 2001 and lower than the present level in Norway. This difference may partly be explained by CAM being reportedly introduced in hospitals later in Denmark. In both countries acupuncture is the dominant reported therapy. The main motive in Denmark for starting up the treatment was scientific evidence, followed by treatment without side effects and interest from the employees. In Denmark no one stated pain relief as a motive, while in Norway this was the third highest ranked reason. We have no explanation for that, although we know that acupuncture is widely used for pain relief, but it might be that it is included in the other motives given. The Danish health personnel inform patients about CAM to a lesser degree than their colleagues in Norway. This could be due to later introduction of CAM in Danish hospitals, and the health personnel could have a suboptimal level of knowledge about possible effects/side effects. The lower level of CAM offered at private hospitals was expected on the basis of more specialized patient groups and the different financial structure of private hospitals. The baseline data both for Norway (from 2001) and Denmark (this study) shows that acupuncture was the dominating CAM modality offered at the hospitals. Among CAM treatments, acupuncture is leading in Norway and reflexology in Denmark in terms of use among the general population [22]. Acupuncture is the only modality that has its own organization only for officially approved health personnel. This might explain why acupuncture is introduced first to hospitals and offered to that extent.

### More positive attitudes towards CAM

The development of CAM in Norwegian and Danish hospitals possibly gives us a signal about a shift in attitude regarding CAM within the conventional health care system. One could speculate that both hospitals and health authorities have changed a former hostility towards CAM to acknowledging its existence and even establishing cooperation with CAM practitioners, with or without a health care background, at the hospitals. This could indicate an increased institutional acceptance of the possible effectiveness/efficacy of some therapy modalities. This acceptance coincides with political initiatives to fund a CAM research center in Norway, funding of CAM research in Denmark and CAM information centres in both countries. The relatively rapid progress in Norway shows how fast treatments mostly considered as not being evidence-based can be implemented in conventional medicine. We expect the same changes to happen in Denmark. This could indicate that the health personnel's main motives for introducing the modalities - "interest from the employees, treatment without side effects, interest from patients" - are stronger than arguments based on evidence/no evidence and arguments based on lack of knowledge about safety. These arguments follow the societal tendencies among patients toward an extensive demand of more natural-based therapies, less medication and more influence on ones own health. In addition, many physicians seek alternatives in their treatment of chronically ill patients and find pharmaceutical treatment alone unsatisfactory [8].

### Comparison with other studies

The Norwegian situation is similar to the results found in Switzerland [14]. Acupuncture is the modality mostly offered in both countries. While more than 1/3 of hospitals in both countries offer more than one therapy form, the specific modalities differ. In addition to acupuncture, patients in Switzerland were offered TCM, homeopathy, neural therapy, herbal medicine, reflexology, manual therapies, packs, and anthroposophic medicine. The results from Denmark show a pattern similar to the Norwegian situation in 2001. We would not be surprised if the further development in Denmark turns out to be similar to Norway. We have not found other comparable studies at the hospital level, but there are studies from Germany showing the use of CAM in departments of obstetrics [23,24]. As in the present study, acupuncture was most commonly used. In addition, the departments of obstetrics offered homeopathy and aromatherapy. In Washington State hospices in USA, CAM is widely offered with massage, music therapy and energy healing being the "most popular therapies" [25]. These hospices offer inpatient and outpatient care. Hospices and hospitals are not necessarily the same, and the results are not fully comparable, but these studies all show a substantial offering of CAM in both in- and outpatients units.

## Conclusion

The extent of CAM being offered at hospitals has increased substantially in Norway during the first decade of the 21^st ^century and the results from Denmark is similar to the results from Norway in 2001 [7]. The present study is based on the medical director's definition of CAM as well as his/her overview of CAM activities going on at the different hospital units. A next step would be to ask about CAM offered on a department level to achieve more in-depth knowledge about the use of CAM within the Norwegian and Danish health care systems. Interesting questions to study is also whether there is collaboration between health care providers and CAM practitioners and what the experiences are.

## Competing interests

The authors declare that they have no competing interests.

## Authors' contributions

LJS has been the project leader. VF developed the questionnaire and the initial idea of the study. VF and LJS developed the interview guide. LS and SLC have done the data collection for the Danish data. LJS drafted the manuscript but LS, LL, and VF has contributed with major improvements, and LN and SLC have contributed with their comments and suggestions. All authors but LL read and approved the final manuscript.

## Pre-publication history

The pre-publication history for this paper can be accessed here:

http://www.biomedcentral.com/1472-6882/11/4/prepub

## Supplementary Material

Additional file 1 **Questionnaire**. The questionnaire used in the study. Click here for file
