# Supplementary material for: Use of complementary and alternative medicine at Norwegian and Danish hospitals
Source: BMC Complement Altern Med. 2011 Jan 18;11:4. doi: 10.1186/1472-6882-11-4 (PMC3033860; doi:10.1186/1472-6882-11-4)
Supplement: Additional file 1 — Questionnaire. The questionnaire used in the study. [file 1472-6882-11-4-S1.DOC]

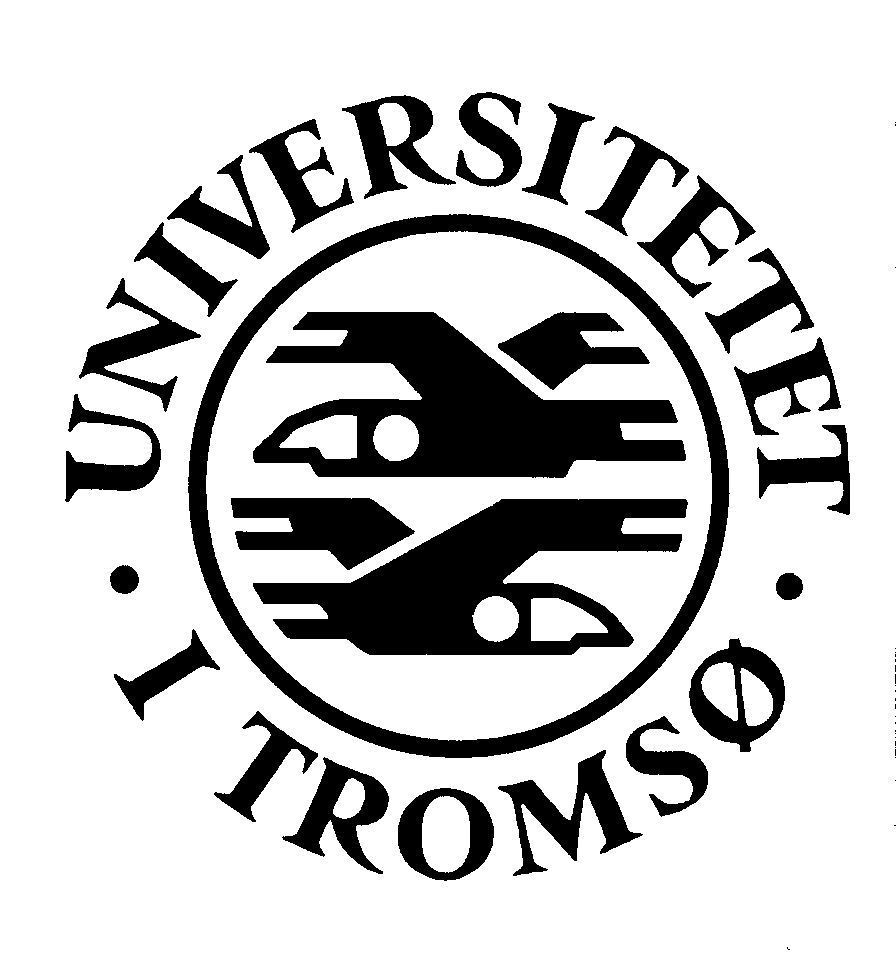


####

Use of alternative treatments at:

The following alternative treatments are in use at our hospital:

No alternative treatments are in use:

Acupuncture

Contact person:_________________________

Homeopathy

Contact person:_________________________

Reflexology

Contact person:_________________________

Herb medicine

Contact person:_________________________

Alternative diet

Contact person:_________________________

Other alternative method

Specify:_____________________________

Contact person:_________________________

The form should be returned to:

Nasjonalt Forskningssenter innen Komplementær og Alternativ Medisin

Universitetet i Tromsø

9037 TROMSØ

Faks: 77646866

E-post: nafkam@fagmed.uit.no
